# Efficacy and safety of onasemnogene abeparvovec for the treatment of patients with spinal muscular atrophy type 1: A systematic review with meta-analysis

**DOI:** 10.1371/journal.pone.0302860

**Published:** 2024-05-07

**Authors:** Brígida Dias Fernandes, Bárbara Corrêa Krug, Fernanda D’Athayde Rodrigues, Hérica Núbia Cardoso Cirilo, Stéfani Sousa Borges, Ida Vanessa D. Schwartz, Livia Fernandes Probst, Ivan Zimmermann

**Affiliations:** 1 Hospital Alemão Oswaldo Cruz, Unidade de Avaliação de Tecnologias em Saúde, São Paulo, SP, Brazil; 2 Instituto Capixaba de Ensino, Pesquisa e Inovação em Saúde (ICEPi), Vitória, ES, Brazil; 3 Secretaria Estadual da Saúde do Rio Grande do Sul, Porto Alegre, RS, Brazil; 4 Federal University of Rio Grande do Sul, Porto Alegre, RS, Brazil; 5 Núcleo de Avaliação de Tecnologias em Saúde do Hospital das Clínicas da Universidade Federal de Goiás/Ebserh, Goiânia, GO, Brazil; 6 Genetics Department, Federal University of Rio Grande do Sul, Porto Alegre, RS, Brazil; 7 Departamento de Saúde Coletiva, Faculdade de Ciências da Saúde, University of Brasilia, Brasília, DF, Brazil; Fondazione Policlinico Universitario Gemelli IRCCS, ITALY

## Abstract

**Background:**

Onasemnogene abeparvovec has been approved for the treatment of spinal muscular atrophy 5q type 1 in several countries, which calls for an independent assessment of the evidence regarding efficacy and safety.

**Objective:**

Conduct a meta-analysis to assess the efficacy and safety of onasemnogene abeparvovec in patients diagnosed with SMA type 1, based on the available evidence.

**Methods:**

This article results from searches conducted on databases up to November 2022. Outcomes of interest were global survival and event-free survival, improvement in motor function and treatment-related adverse events. Risk of bias assessment and certainty of evidence were performed for each outcome. Proportional meta-analysis models were performed when applicable.

**Results:**

Four reports of three open-label, non-comparative clinical trials covering 67 patients were included. Meta-analyses of data available in a 12-month follow-up estimate a global survival of 97.56% (95%CI: 92.55 to 99.86, *I*^2^ = 0%, n = 67), an event-free survival of 96.5% (95%CI: 90.76 to 99.54, *I*^2^ = 32%, n = 66) and a CHOP-INTEND score ≥ 40 points proportion of 87.28% (95%CI: 69.81 to 97.83, *I*^2^ = 69%, n = 67). Proportion of 52.64% (95%CI: 27.11 to 77.45, *I*^2^ = 78%, n = 67) of treatment-related adverse events was estimated.

**Conclusion:**

The results indicate a potential change in the natural history of type 1 SMA, but the methodological limitations of the studies make the real extent of the technology’s long-term benefits uncertain.

## 1. Introduction

Spinal muscular atrophy 5q (SMA) is a genetic disorder, of an autosomal recessive inheritance pattern, characterized by progressive loss of muscle strength caused by degeneration of neurons in the anterior horn of the spinal cord [1]. Its classical form is caused by biallelic variants in survival motor neuron 1 (*SMN1*), normally deletions of exons 7 and 8 [2]. SMA is a rare disorder, with an estimated prevalence of 1–2 per 100,000 people and an incidence of 8 per 100,000 live births, considering all SMA types [3]. Research shows that it is one of the leading genetic causes of infant mortality [4].

SMA has a heterogeneous clinical presentation, showing variability with regard to onset age, clinical symptoms and progression rate. There are four types, which range from severe (type 1, symptoms in infancy and never able to sit) to mild (type 4, adult onset walking difficulties), with the former being the most prevalent [5]. The clinical variability is partly attributed to the number of copies of the *SMN2* gene in patients. However, there is an increasing body of evidence suggesting that additional factors play a role in the manifestation of the disease [6].

Given its progressive development, patients require several forms of specialized care covering support therapies by multiprofessional teams; these include neurologic, respiratory, physiotherapist, nutritional, and orthopedic assistance, among other types. Specific pharmacological treatments currently available are nusinersen and risdiplam, which promote and increase in the production of full length functional SMN protein through modification of *SMN2* [7]. More recently, the use of onasemnogene abeparvovec (Zolgensma®; Novartis Gene Therapies), a gene therapy that replaces the *SMN1* gene to allow for restored expression of the full length SMN protein [8], was approved in several countries. It is made up of a non-replicating recombinant viral vector (adeno-associated vector of serotype 9 or AAV9) modified to contain the cDNA of the human *SMN1* gene, administered intravenously by a single infusion [8]. Onasemnogene abeparvovec is indicated for the treatment of SMA patients with bi-allelic mutations in the *SMN1* gene only for those under 2 years of age, in the United States [9] and Brazil [10]. The indication in Europe [11], Canada [12] and Brazil [10] includes the clinical diagnosis of SMA type 1 and patients with up to 3 copies of *SMN2*.

Previous systematic reviews have sought to assess the efficacy and safety of onasemnogene abeparvovec in relation to other technologies but have proved inconclusive with regard to some outcomes due to the impossibility of a connected network analysis and lack of data from all the studies available on the subject [13, 14]. Other systematic reviews have focused on real-world studies, without statistical synthesis of the data [15], and on evaluating the efficacy—based only on the assessment of motor function—and safety of onasemnogene abeparvovec in patients with SMA [16, 17]. Because it is an innovative and costly technology [18], an independent systematic review on the effectiveness and safety of onasemnogene abeparvovec in the main outcomes (survival, motor function and adverse events) is necessary to inform decision makers about the value of this technology considering the quality of clinical trials and the certainty of evidence. Therefore, this systematic review has sought to assess available evidence on the efficacy and safety of onasemnogene abeparvovec in SMA type 1 patients.

## 2. Methodology

### 2.1 Protocols and registries

To answer the question “Is onasemnogene abeparvovec effective and safe for treating SMA type 1 patients?”, a systematic review of the literature was carried out and its protocol registered previously on PROSPERO (CRD42022302016). The Preferred Reporting Items for Systematic Reviews and Meta-Analyses (PRISMA) checklist was used to guide the review report (S1 File) [19].

### 2.2 Eligibility criteria

We considered eligible for this study clinical trials (controlled and uncontrolled, randomized and nonrandomized) performed with individuals diagnosed with SMA type 1, aged up to 2 years, who did not require permanent invasive ventilation and were given onasemnogene abeparvovec. Eligible comparatives were nusinersen and risdiplam, as well as support measures, placebo, and no intervention.

Global survival, event-free survival, achievement of motor milestones, and treatment-related adverse events were considered primary outcomes of this review. Event-free survival refers to the occurrence of death or permanent mechanical ventilation in accordance with criteria defined by each research study. As for achievement of motor milestones, studies that assessed motor function via validated and internationally endorsed scales Bayley-III Scales of Infant and Toddler Development (BSID) and Children’s Hospital of Philadelphia Infant Test of Neuromuscular Disorders (CHOP INTEND), as well as the WHO Multicenter Growth Reference Study, were considered eligible. Secondary outcomes of interest were serious adverse events not necessarily related to the treatment, and improvement in motor function and quality of life.

Studies conducted with patients who were diagnosed with other neuromuscular disorders in addition to SMA, namely, brain paralysis, congenital muscular dystrophy, juvenile myasthenia gravis, congenital heart disease and muscular dystrophy, among others, were excluded from this review.

### 2.3 Data sources and search strategies

The search for eligible studies was performed by five reviewers (BF/BK/HC/SB/FR) on databases MEDLINE (via PubMed), Embase, LILACS, Cochrane Library, Clinicaltrials.gov and WHO International Clinical Trials Registry Platform (ICTRP), and covered all studies published up to November 2022 in any language. Descriptors and their respective synonyms related to intervention (onasemnogene abeparvovec), comparatives (nusinersen and risdiplam), and clinical condition (SMA type 1) were combined to produce a more refined set of results. Manual search was carried out on the references listed in the studies selected for review to locate additional eligible studies. To ensure the review’s reproducibility and future update, the terms used and the complete search strategies are described in the S1 File.

### 2.4 Selection process

After the removal of duplicates, selection of titles and abstracts (Phase 1) and subsequent full reading of texts (Phase 2) were carried out independently and paired by two sets of reviewers (BK/HC/SB. and BF/FR). Divergences were settled through consensus among researchers or by two other reviewers (I.Z/LP). Removal of duplicates and selection of studies were conducted with the use of the Rayyan [20] web application.

### 2.5 Data collection process

Independent and paired data extraction by two researchers was conducted via a form created and previously tested on Excel 2013. Extracted data referred to studies’ characteristics (author and publication year, name of clinical trial, country in which it was conducted, sponsors, study design, follow-up time, inclusion criteria, randomization and allocation concealment); population (total number of individuals, baseline weight; gestational, at diagnosis and at administration of technology ages; support treatments); technologies (intervention dosage); and outcomes of interest. Divergences related to the extracted data were settled through consensus or debate among reviewers. Data not given in textual form were extracted from graphs or appendices by a reviewer via WebPlotDigitizer (https://automeris.io/WebPlotDigitizer/).

### 2.6 Risk of bias assessment

For randomized studies, we used Cochrane’s risk-of-bias (RoB) tool, which analyzes risk of bias based on available data on the following domains: random sequence generation; allocation concealment; masking (blinding) of participants and research team; masking (blinding) in outcome assessment; incomplete outcome data; selective outcome reporting; and other sources of bias. The Risk of Bias in Non-Randomized Studies–of Interventions (ROBINS-I) tool was, in turn, used to assess risk of bias in nonrandomized studies. This tool considers each study as an attempt to imitate a hypothetical pragmatic randomized trial and covers seven different domains through which bias can be introduced [21]. Given that, due to the characteristics of the health condition under assessment, the intervention studies designed are single-arm, assessment criteria of risk of bias were thus adapted: each of the seven domains was regarded as low risk of bias once it was acknowledged that a high-quality randomized study would reveal the same features. If, however, a study distanced itself from the RCT hypothesized early on, the domain would be regarded as high risk of bias. It should be noted that adaptations in risk-of-bias tools are often conducted to meet the specificities of studies under review, since no tools have yet been specifically designed to assess risk of bias in single-arm intervention trials [22–24].

Outcomes of interest were individually assessed in each study and ranked as ‘critical’, ‘serious’, ‘moderate’ and ‘low’ risk of bias, as well as ‘information not available’. Domains considered were the following: confounding bias, selection bias, intervention bias, detour intervention bias, information bias, outcome measure bias and outcome reporting bias. Researchers collectively discussed each domain until reaching a consensus.

### 2.7 Effect measures

For dichotomous outcomes, absolute and relative frequencies (and respective 95% confidence intervals) were presented for studies that defined a time-invariant assessment. For continuous outcomes, we considered mean scores yielded in motor function improvement via motor development scales.

Safety outcomes, which refer to the occurrence of adverse events, general and serious, associated or not with treatment, were expressed in absolute and relative frequencies.

### 2.8 Synthesis methods

The research design established a narrative synthesis and presentation of results from reviewed studies in structured tables containing brief statistics for each result of interest. If at least two of the studies reviewed were sufficiently homogenous, results would be gathered in a meta-analysis. A fixed-effect model was assigned for minimal heterogeneity and a random-effect model for substantial heterogeneity. Moderate heterogeneity was established in the case of a 50% *I*^2^ threshold, also considering the *p*-value [25]. In the absence of comparative studies, the possibility of performing proportion meta-analysis models was considered for evidence synthesis of dichotomous outcomes of efficacy and safety with the same follow-up time for all the studies reviewed (12 months); this entailed the use of variance inversion and arcsine transformation. Sensitivity analyses using other transformations, such as log and logit transformation, were also conducted [26]. As outlined in the protocol, in case of sufficiently available data, a sub-group analysis would be carried out based on the age of patients being treated with onasemnogene abeparvovec. Meta-analysis models were designed via the use of R and statistical packages metafor and metaprop. The complete codes and databases used in the meta-analyses are available in public repository GitHub (URL omitted to preserve authors’ identities).

### 2.9 Risk of bias report

It was assessed whether each relevant result was measured, analyzed and reported by comparing published articles with studies’ available registries or protocols. In case of missing data, authors would be contacted. In addition, monitoring of at least 80% of participants included in each study was examined to determine the robustness of results. The research design also included assessment of risk of publication bias, when applicable, via funnel plot and asymmetry regressions (Egger’s test) in case 10 or more studies were included.

### 2.10 Certainty of evidence assessment

Certainty of evidence for each outcome was collectively verified by reviewers and categorized as high, moderate, low and very low based on the Grade of Recommendations, Assessment, Development and Evaluations (GRADE) approach. Summary of Findings (SoF) tables were produced via GRADEpro GDT [27].

## 3. Results

### 3.1 Selection of studies

**Fig 1:**
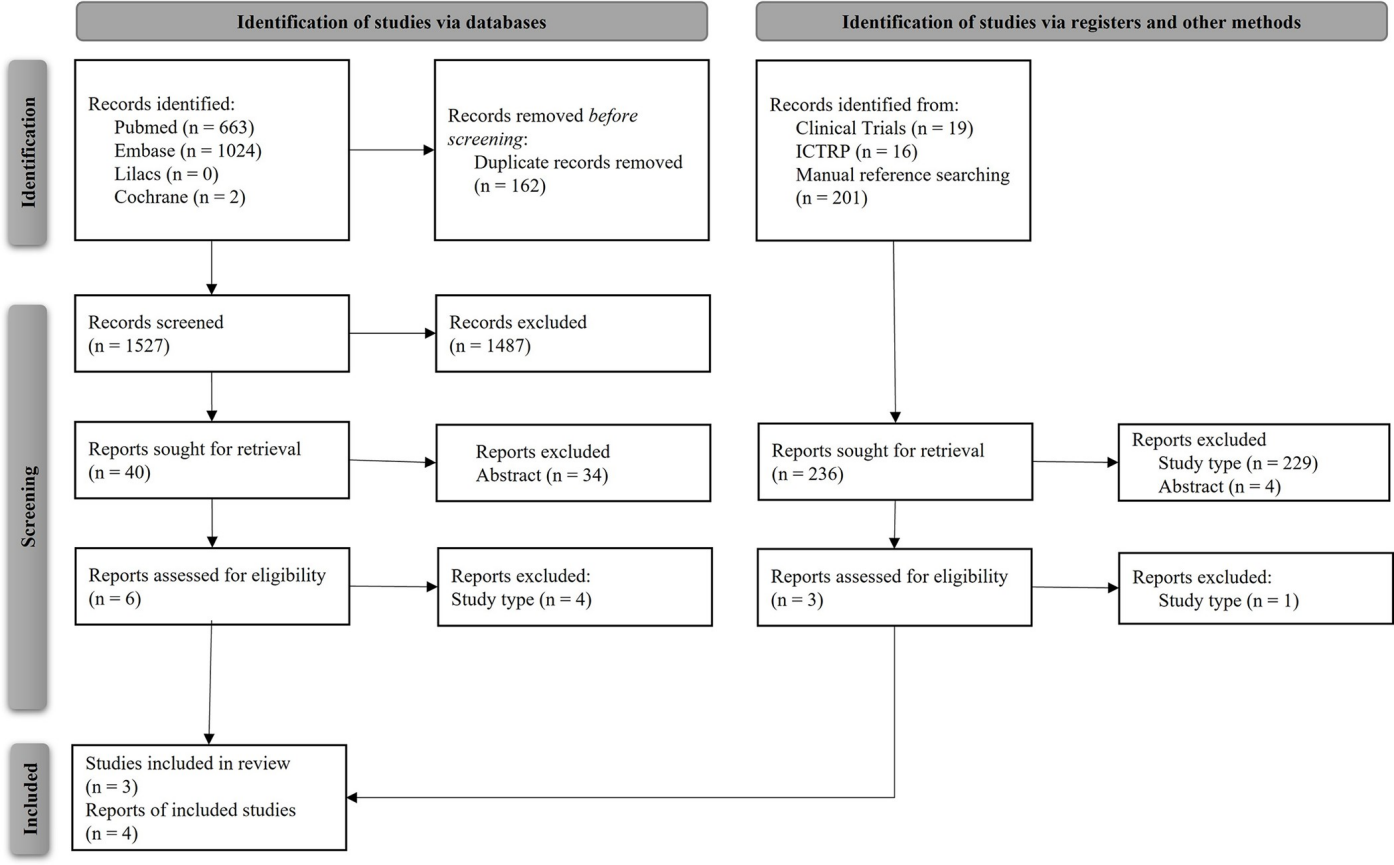
Flowchart of studies’ selection. Adapted from: Page et al., 2021 [19]. Available at: http://www.prisma-statement.org/.

Database searches yielded 1,689 reports; of these, 162 were duplicates and 1,487 did not meet inclusion criteria, being therefore excluded. In parallel, 236 references were identified on clinical trials registries database and through manual searches. Following the application of eligibility criteria to all complete studies accessed, 38 were excluded (see S1 File), which resulted in the selection of four reports, related to three studies, for inclusion in this systematic review (Fig 1).

### 3.2 Characteristics of the reviewed studies

Four reports from three open-label, nonrandomized clinical trials were selected to comprise this review (Table 1). Outcomes related to quality of life and to reduction in the need for ventilatory support were not verified in these studies.

**Table 1:** Characterization of studies included in the systematic review.

Characteristics	Clinical trial		
	START	STR1VE-US	STR1VE-EU
Reference	Mendell et al., 2017 [28]; Mendell et al., 2021 [29]	Day et al., 2021 [30]	Mercuri et al., 2021 [31]
Country	United States	United States	Italy, UK, Belgium and France
Location	Unicentric	Multicentric	Multicentric
Study design	Single-arm open-label, phase 1	Single-arm open-label, phase 3	Single-arm open-label, phase 3
Participants treated with intervention	12 patients with SMA type 1 and onset of symptoms up to 6 months of age, homozygous deletion of *SMN1* exon 7 and two copies of *SMN2*; after 14 months, only 10 of these patients were monitored	22 patients with SMA type 1 and aged < 6 months, with biallelic *SMN1* mutation (deletion or sporadic mutations) and two or more copies of *SMN2*	33 patients with SMA type 1 and aged < 6 months, with biallelic *SMN1* mutation (deletion or sporadic mutations) and one or two copies of *SMN2*
Baseline weight	5.7 (3.6–8.4) †	5.8 kg (5.1–6.5)‡	5.8 kg (5.1–6.6) ‡
Gestacional age	Not informed	39.0 weeks (39.0–39.0)‡	39.0 weeks (38.0–40.0)‡
Participants’ age at diagnosis	60 days (0–136)†	67.0 days (56.0–126.0)‡	76.0 days (59.0–105.0)‡
Participants’ age during administration of intervention	3.4 months (0.9–7.9)†	3.5 months (2.7–5.3)‡	4.1 months (3.0–5.2)‡
Participants’ age at end of follow-up	Average of 5 years	Follow-up until 18 months	Follow-up until 18 months
Intervention	Single-dose intravenous infusion containing 1,1 × 10^14^ viral genomes/kg of onasemnogene abeparvovec	Single-dose intravenous infusion containing 1,1 × 10^14^ viral genomes/kg of onasemnogene abeparvovec	Single-dose intravenous infusion containing 1,1 × 10^14^ viral genomes/kg of onasemnogene abeparvovec
Support treatment	Enteral feeding via gastrostomy or nasogastric tube and non-invasive ventilatory support	None	Enteral feeding via gastrostomy or nasogastric tube and non-invasive ventilatory support for at least 12 hours daily
Reported outcomes of interest	Global survival, mechanical-ventilation-free survival, improvement in motor function and treatment-related adverse events	Global survival, mechanical-ventilation-free survival, improvement in motor function, treatment-related adverse events	Global survival, mechanical-ventilation-free survival, improvement in motor function, treatment-related adverse events
Financing sources	Novartis Gene Therapies, Inc.	Novartis Gene Therapies, Inc.	Novartis Gene Therapies, Inc.
Bias from unreported results*	Not identified	Not identified	Not identified

* Assessment based on the comparison between published results and protocols of each study.

† Mean (aamplitude).

‡ Median (interquartile range).

### 3.3 Risk of bias of reviewed studies

START, STR1VE-US and STR1VE-EU studies were categorized as high risk of bias in the assessment of outcomes of interest, particularly due to the assessment of domains of confounding factors (D1) and outcome measurement (D6).

**Fig 2:**
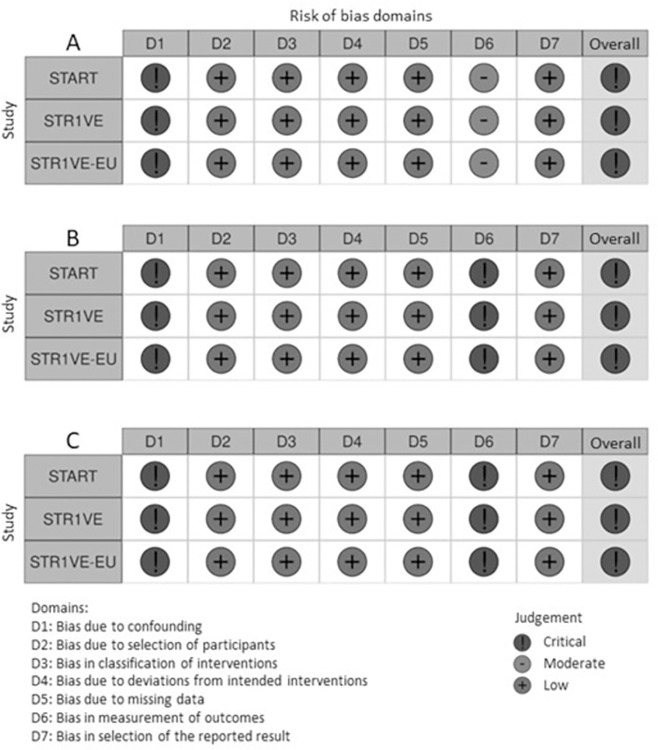
Risk of bias assessment, outcomes of global survival and mechanical-ventilation-free survival (A), improvement in motor function (B) and treatment-related adverse events (C).

For outcomes related to global survival and event-free survival, improvement in motor function and safety (Fig 2), confounding factors were categorized as high risk of bias due to potential confounding factors of the intervention’s effect, possibly not adjusted by an appropriate method of control analysis, both in the baseline and in studies’ monitoring. Judgement of all domains with justifications may be viewed in the S1 File.

### 3.4 Efficacy outcomes

#### 3.4.1 Global survival and event-free survival

All 12 patients (100%) were alive and free from ventilatory support at 20 months of age in the START study [28] and the 10 patients (100%) included in the five-year extension were alive and did not require ventilatory support at the end of follow-up [29]. Of the 22 patients included in the STR1VE-US study, 20 (91%, 95%CI: 79–100%) did not require permanent ventilatory support and 19 (86%) completed the study [30]. In the STR1VE-EU study, global survival was reached by 31 (97%, 95%CI: 91–100%) of the 32 patients in the Intent-to-treat (ITT) population [31].

**Fig 3:**
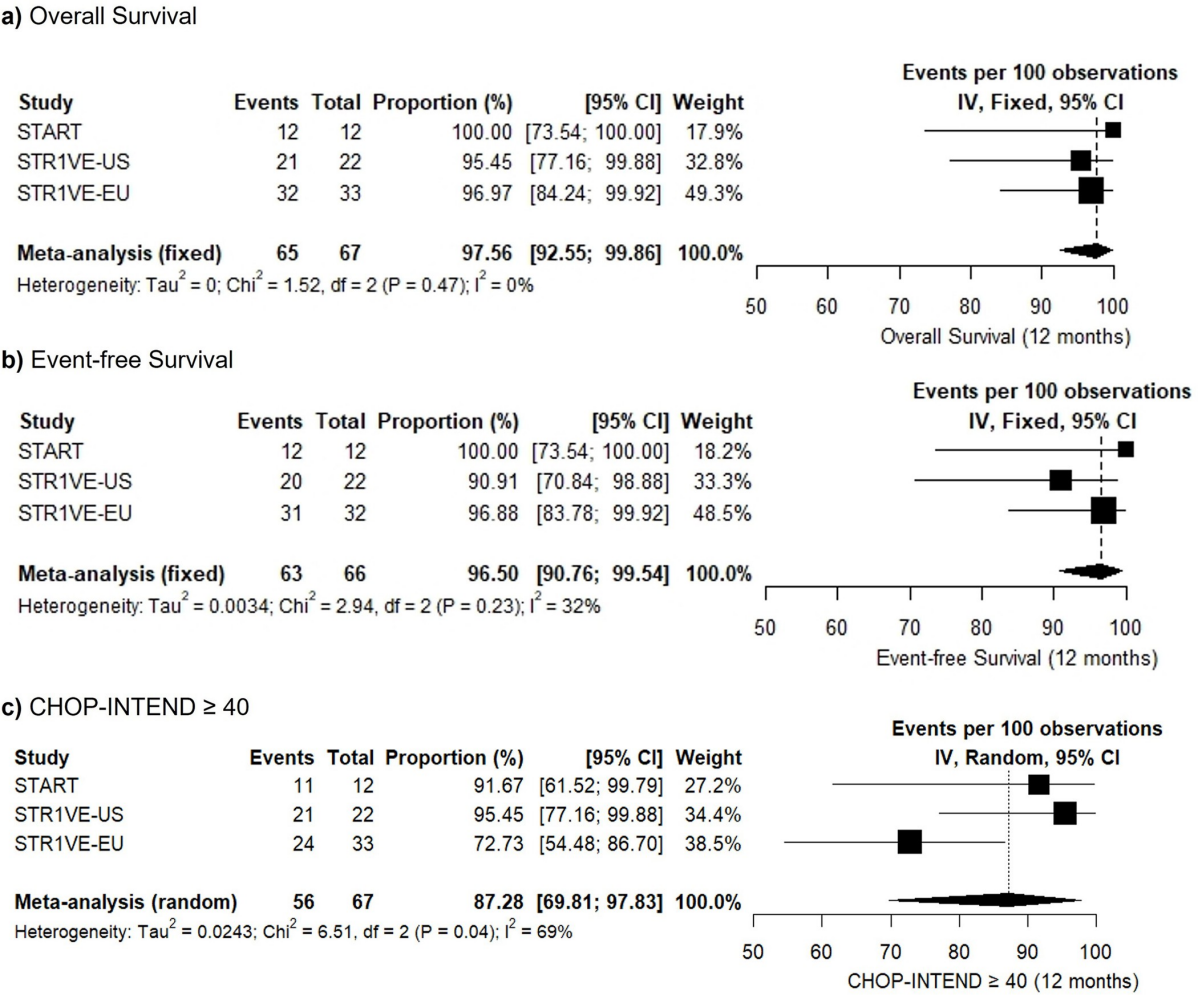
Meta-analysis of a) overall survival, b) event-free survival and c) CHOP-INTEND ≥ 40 following administration of onasemnogene abeparvovec in SMA type 1 patients.

Available data enabled proportion meta-analysis models to be conducted. During the 12-month follow-up, global survival was estimated at 97.56% (95%CI: 92.55–99.86, *I*^2^ = 0%, n = 67) and event-free survival at 96.5% (95%CI: 90.76–99.54, *I*^2^ = 32%, n = 66). As shown in Fig 3, inconsistency was not a major factor in these outcomes. Such effect estimates at 12 months were consistent with follow-up times of up to 18 months.

Sensitivity analysis via log and logit transformations did not impact on individual estimates, albeit indicated higher imprecision of confidence intervals of the combined estimate.

#### 3.4.2 Improvement of motor function

In the START study, two patients began to walk unsupported [28], whereas in the STR1VE study only one achieved this motor milestone [30, 31]. All motor milestones remained during START’s extension, with no regression or loss of function [29].

Meta-analysis of the data provided by studies estimated a proportion of patients reaching a CHOP-INTEND score ≥ 40 of 87.28% (95%CI: 69.81–97.83, *I*^2^ = 69%, n = 67). Unlike in survival outcomes, this finding shows a major heterogeneity, particularly related to the estimates yielded by the STR1VE-EU study (Fig 3).

### 3.5 Safety outcomes

#### 3.5.1 Adverse events

In the START study, 10 (83%) patients experienced at least one serious adverse event, with three patients undergoing events directly related to the treatment (increase in ALT and AST) [28]. During the extension, only serious adverse events were reported, none of which related to the treatment [29]. All 22 patients of the STR1VE-US study showed adverse events, of which 10 (45%) had some form of serious adverse event and 3 (14%) had serious treatment-related adverse events (two had an increase in ALT and AST and another had hydrocephalus) [30]. In the STR1VE-EU study, at least one adverse event was reported by 32 (97%) of 33 patients and six (18%) had serious treatment-related adverse events. One reported death was unrelated with the intervention in question [31]. A detailed description of treatment-related adverse events can be consulted in the S1 File.

**Fig 4:**
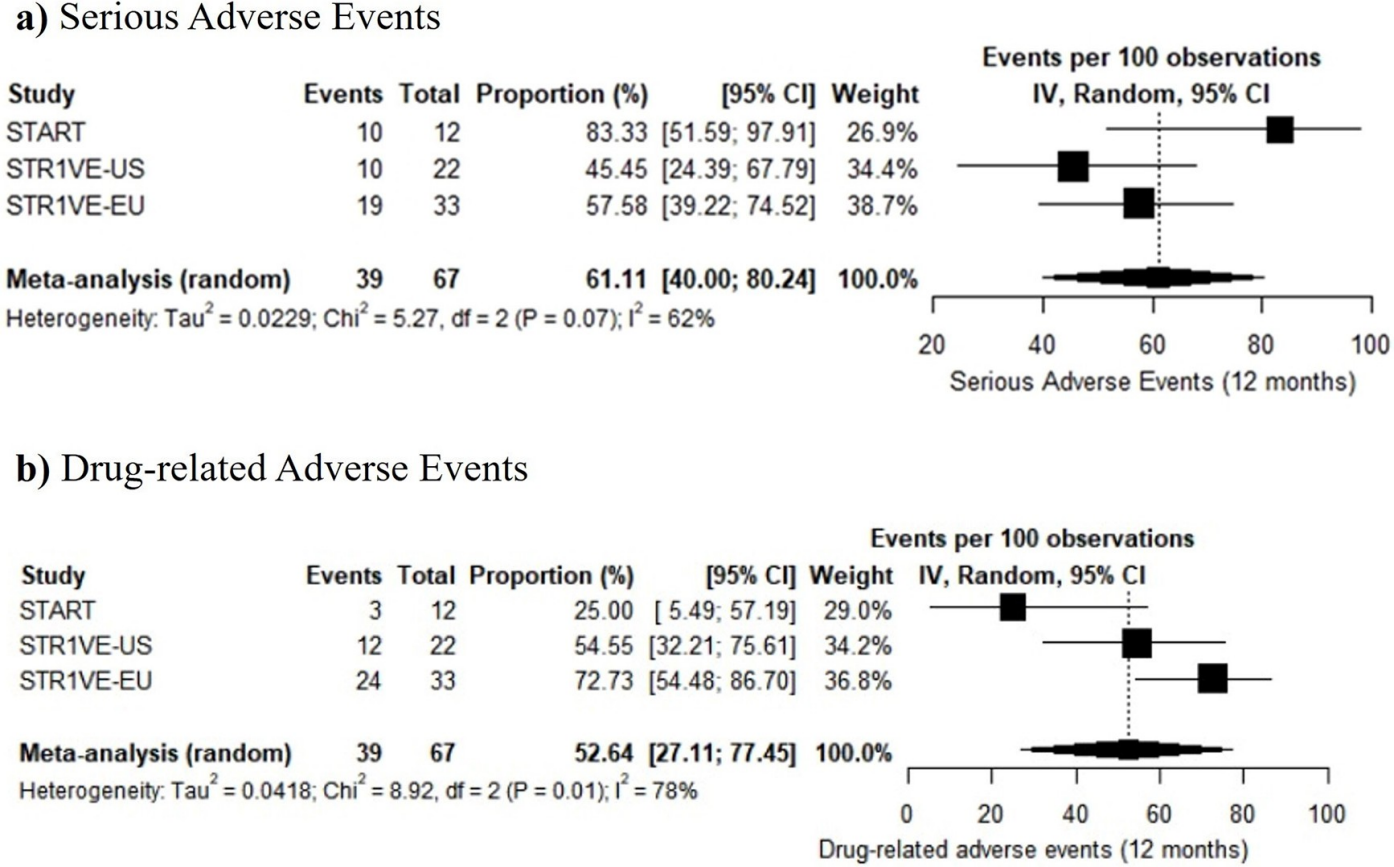
Meta-analysis of a) serious adverse events and b) drug-related adverse events following administration of onasemnogene abeparvovec in SMA type 1 patients.

Meta-analysis models estimated a 61.11% (95%CI: 40.00–80.24, *I*^2^ = 62%, n = 67) proportion of serious adverse events and a 52.64% (95%CI: 27.11–77.45, *I*^2^ = 78%, n = 67) proportion of treatment-related adverse events (Fig 4). Models revealed a major inconsistency in the analysis of these events, possibly linked to START’s sample size.

The complete data of extractions of all outcomes are available in S1 File tables.

### 3.6 Certainty of evidence

Table 2 summarizes findings and certainty of evidence with the GRADE assessment of critical outcomes. Given the small number of studies reviewed, publication bias was not assessed via a funnel plot and asymmetry regression. Considering the different follow-up times of global survival and mechanical-ventilation-free survival, these outcomes are presented separately. Certainty of evidence was characterized as very low quality for all outcomes, mainly due to downgrading by two levels resulting from studies’ risk of bias assessment.

**Table 2:** Profile of certainty of evidence in the treatment with onasemnogene abeparvovec in patients of up to 2 years of age diagnosed with SMA type 1.

Assessment of certainty of evidence							Observed effects	Certainty of evidence	Importance
No. of studies	Research design	Risk of bias	Inconsistency	Indirect evidence	Imprecision	Other remarks			
**Global survival (follow-up: 12 months; assessed with: proportion of deaths)**									
3 (67 patients)	nonrandomized clinical trial	very serious^a,b^	not serious	not serious	serious^d^	none	The 12-month follow-up yielded a global survival estimate of 97.56% (95%CI: 92.55 to 99.86, *I*^2^ = 0%)	⨁◯◯◯ Very low	CRITICAL
**Event-free survival (follow-up: 12 months; assessed with: proportion of death or permanent ventilation)**									
2 (66 patients)	nonrandomized clinical trial	very serious^a,b^	not serious	not serious	serious^d^	none	The 12-month follow-up yielded a global survival estimate of 96.5% (95%CI: 90.76 to 99.54, *I*^2^ = 32%)	⨁◯◯◯ Very low	CRITICAL
**Event-free survival (follow-up: 120 months on average)**									
1 (10 patients)	nonrandomized clinical trial	very serious^a,b^	not serious	not serious	serious^d^	none	During START’s extension, all patients survived 5.0 years on average (4.6–5.6) without the need for permanent mechanical ventilation.	⨁◯◯◯ Very low	CRITICAL
**Safety (treatment-related adverse events) (follow-up: 12 months)**									
3 (67 patients)	nonrandomized clinical trial	very serious^a,b^	serious^c^	not serious	serious^d^	none	The 12-month follow-up yielded a global survival estimate of 52.64% (95%CI: 27.11 to 77.45, *I*^2^ = 78%).	⨁◯◯◯ Very low	CRITICAL
**Motor response (follow-up: 12 to 24 months; assessed with: CHOP INTEND ≥ 40)**									
3 (67 patients)	nonrandomized clinical trial	very serious^b,e^	serious^c^	not serious	serious^d^	none	The 12-month follow-up yielded a global survival estimate of 87.28% (95%CI: 69.81 to 97.83, *I*^2^ = 69%, n = 67).	⨁◯◯◯ Very low	IMPORTANT

CI: confidence interval.

Explanations: a. Absence of comparison, randomization or adjustments for confounding factors. b. Downgraded in two levels, justified by risk of bias assessment via Robins-I. c. Large confidence interval. d. Sample size considered small. e. Absence of comparison, randomization, blinding, adjustments for confounding factors and subjectivity in the application of instruments to assess motor scores.

Unlike other outcomes, the inconsistency domain was not downgraded as regards mechanical-ventilation-free survival in 120-month follow-ups (START’s extension study), since no heterogeneity was detected in any level of the report’s assessment. On the other hand, the imprecision domain was downgraded for all outcomes in view of studies’ very small sample size.

## 4. Discussion

Over the course of this review, it became clear that there are still few studies focused on assessing the role of onasemnogene abeparvovec in the treatment of SMA type 1. Even after an extensive search in databases and clinical trials registries, the review produced only four reports of three open-label, nonrandomized clinical trials, with no active control group and small sample size. The evidence points to the efficacy of onasemnogene abeparvovec in increasing survival in SMA type 1 patients and in promoting motor improvement, hence presenting satisfactory data regarding the safety of patients included in these studies. However, confidence in such evidence is low since assessment is conducted by studies devoid of a control group, in a context marked by treatment options that have also shown efficacy for the same outcomes [7, 32].

A cohort of SMA’s natural history has shown that the median survival time reached 8 months for patients with two copies of *SMN2* [33]. Studies on onasemnogene abeparvovec conducted with patients who had two copies of *SMN2* yielded, in turn, an estimated global survival of 97.56%, and event-free survival of 96.5%, during the 12-month follow-up. Motor function decreased progressively in the cohorts of SMA’s natural history [33, 34]. Conversely, the meta-analysis of data available in a 12-month follow-up estimated a proportion of CHOP-INTEND score ≥ 40 points of 87.28%, similar to the findings of other systematic reviews [16, 17].

It should be noted that the observed effects of the technology in question may have been influenced by patients’ age in the administration of onasemnogene abeparvovec, since the studies included in this review were performed with patients for whom the highest range of age at administration of medication was 0.9–7.9 months. In the event that early administration of gene therapy produces better results, the SPR1NT study assessed the application of onasemnogene abeparvovec in patients with risk of developing SMA type 1, treated prior to 6 weeks of age and the onset of symptoms [35]. Results showed that all the patients survived without permanent ventilation at 14 months according to protocol, seated independently for ≥ 30 seconds, and nine (64%) walked independently according to BSID criteria [35]. These findings draw attention to the issue of early diagnosis via newborn screening and the ideal moment to administer onasemnogene abeparvovec [36]. With regard to safety, the results of clinical trials showed mainly an increase in liver transaminase levels, which made it necessary to use prednisolone combined with onasemnogene abeparvovec [28, 30, 31], a recommendation contained in the technology’s package insert.

Recent post-commercialization reports of fatal acute liver failure following the administration of onasemnogene abeparvovec have coincided with the reduction in corticosteroid dosage [37]. Following a general examination of the safety of onasemnogene abeparvovec, which included post-commercialization data, five categories of potential adverse events of special interest (hepatotoxicity, thrombocytopenia, cardiac events, thrombotic microangiopathy and ganglionopathy) were identified, and mitigation strategies proposed for each risk described [38]. Further studies with longer monitoring periods and the assessment of adverse event notifications are required for the safety profile of onasemnogene abeparvovec in this population to be accurately established.

In START’s follow-up, four out of 10 patients of the cohort treatment via onasemnogene abeparvovec were taking nusinersen [29]. This combination of treatments has been reported in real-world studies [39, 40] with a view to maximizing clinical results in patients. These results highlight the need for comparative and combined analyses of the various treatments available to assess individual and combined clinical benefits. Therefore, the studies covered by this review show major methodological limitations, which pose high risk of bias and need to be considered in the interpretation of reported effects. Even though these studies point to the survival of most SMA type 1 patients at 14 and 20 months of age, more proactive clinical care, combined with nutritional and ventilatory support as well as with technologies nusinersen and risdiplam [7, 32], also ensured these patients an increase in survival [41]. Therefore, the presence of a control group is crucial to estimate the real statistical and clinical effect of onasemnogene abeparvovec. On the other hand, support care may not account for improvements in motor function (in decline in SMA’s natural history), but lack of blinding in studies may superestimate subjective data, such as those measured by scale application and observation [42].

Despite such methodological limitations, the decision to incorporate the technology in universal health systems or to foster such a recommendation in clinical guidelines should be taken based on results currently available, for which transparent certainty of evidence becomes crucial. In the present assessment of onasemnogene abeparvovec, certainty of evidence was very low for all outcomes, hence calling for a cautious decision-making process, particularly in the case of cost-effectiveness analyses. In addition to a very high risk of bias, small sample sizes, the absence of a control group that hinders randomization and, consequently, statistical summarization also produce imprecise results. Previous systematic reviews have provided indirect comparisons between onasemnogene abeparvovec and other technologies via techniques such as Matching-Adjusted Indirect Comparison (MAIC) and Simulated Treatment Comparison (STC) and proved inconclusive with regard to some outcomes or identified imprecise results due to large confidence intervals [13, 14]. Even though the use of these statistical methods in decision-making has become increasingly popular [43], indirect comparison between various technologies has proved not suitable due to the lack of a connected network and to a need for patients’ individual data (which are not publicly available). In a similar vein to the primary studies included in this review, studies that conducted indirect comparison analyses between treatments were sponsored by the pharmaceutical industry, hence requiring independent updates.

This study shows a number of strengths, above all the ample search in indexed databases and clinical trials registries. The selection process rigorously followed Cochrane Collaborations’ recommendations with a view to avoiding selection bias through the role of independent reviewers. Nevertheless, reviewers’ interpretation faces some limitations, mostly related to the challenge of summarizing the evidence from single-arm, open-label trials without direct or indirect comparatives. Assessment of quality of evidence may have been influenced by reviewers’ subjective viewpoints, given that the ROBINS-I tool was designed for nonrandomized intervention studies and simply adapted for single-arm studies. Future research should focus on cost-effectiveness analyses to support decision-making on the incorporation of technologies and recommendations in treatment guidelines. Other major outcomes such as quality of life should also be considered, as well as systematic measurement of the occurrence and severity of adverse events.

## 5. Conclusion

SMA type 1 is a rare disease that, if untreated, may lead to severe incapacity and death. This review has shown that, despite improvements in survival and motor function outcomes, all with satisfactory safety profiles, the studies found in the literature were not designed to address the effect of onasemnogene abeparvovec comparatively and in the long term. Such limitations, associated with the characteristics of a rare disease, blur the real extent of the technology’s benefits, hence it is crucial that results be monitored and assessed in the context of clinical practice.

## Supporting information

S1 File (DOCX)
