# Supplementary material for: Efficacy and safety of onasemnogene abeparvovec for the treatment of patients with spinal muscular atrophy type 1: A systematic review with meta-analysis
Source: PLoS One. 2024 May 7;19(5):e0302860. doi: 10.1371/journal.pone.0302860 (PMC11075831; doi:10.1371/journal.pone.0302860)
Supplement: S1 File — (DOCX) [file pone.0302860.s001.docx]

**Supplementary file**

[**Supplementary material 1.** PRISMA 2020 checklist. 2](#_Toc121042818)

[**Supplementary material 2.** Databases and respective search strategies employed, date of search and number of studies retrieved. 5](#_Toc121042819)

[**Supplementary material 3.** Characteristics of excluded studies. 9](#_Toc121042820)

[**Supplementary material 4.** Risk of bias assessment using the ROBINS-I tool for studies by Day et al. (2021); Mercuri et al. (2021) and Mendell et al. (2017). 12](#_Toc121042821)

[**Supplementary material 5.** Clinical trial results for overall survival, event-free survival, motor and other milestones achievements. 14](#_Toc121042822)

[**Supplementary material 6.** Clinical trial results for safety. 16](#_Toc121042823)

[**References** 18](#_Toc121042824)

# **Supplementary material 1.** PRISMA 2020 checklist.

| **Section and Topic** | **Item #** | **Checklist item** | **Location where item is reported** |
| --- | --- | --- | --- |
| **TITLE** | | |  |
| Title | 1 | Identify the report as a systematic review. | 1 |
| **ABSTRACT** | | |  |
| Abstract | 2 | See the PRISMA 2020 for Abstracts checklist. | 2 |
| **INTRODUCTION** | | |  |
| Rationale | 3 | Describe the rationale for the review in the context of existing knowledge. | 2-4 |
| Objectives | 4 | Provide an explicit statement of the objective(s) or question(s) the review addresses. | 4 |
| **METHODS** | | |  |
| Eligibility criteria | 5 | Specify the inclusion and exclusion criteria for the review and how studies were grouped for the syntheses. | 4,5 |
| Information sources | 6 | Specify all databases, registers, websites, organisations, reference lists and other sources searched or consulted to identify studies. Specify the date when each source was last searched or consulted. | 5 |
| Search strategy | 7 | Present the full search strategies for all databases, registers and websites, including any filters and limits used. | Supplementary File |
| Selection process | 8 | Specify the methods used to decide whether a study met the inclusion criteria of the review, including how many reviewers screened each record and each report retrieved, whether they worked independently, and if applicable, details of automation tools used in the process. | 5,6 |
| Data collection process | 9 | Specify the methods used to collect data from reports, including how many reviewers collected data from each report, whether they worked independently, any processes for obtaining or confirming data from study investigators, and if applicable, details of automation tools used in the process. | 6 |
| Data items | 10a | List and define all outcomes for which data were sought. Specify whether all results that were compatible with each outcome domain in each study were sought (e.g. for all measures, time points, analyses), and if not, the methods used to decide which results to collect. | 6 |
|  | 10b | List and define all other variables for which data were sought (e.g. participant and intervention characteristics, funding sources). Describe any assumptions made about any missing or unclear information. | 6 |
| Study risk of bias assessment | 11 | Specify the methods used to assess risk of bias in the included studies, including details of the tool(s) used, how many reviewers assessed each study and whether they worked independently, and if applicable, details of automation tools used in the process. | 6,7 |
| Effect measures | 12 | Specify for each outcome the effect measure(s) (e.g. risk ratio, mean difference) used in the synthesis or presentation of results. | 7 |
| Synthesis methods | 13a | Describe the processes used to decide which studies were eligible for each synthesis (e.g. tabulating the study intervention characteristics and comparing against the planned groups for each synthesis (item #5)). | 7,8 |
|  | 13b | Describe any methods required to prepare the data for presentation or synthesis, such as handling of missing summary statistics, or data conversions. | 7,8 |
|  | 13c | Describe any methods used to tabulate or visually display results of individual studies and syntheses. | 7,8 |
|  | 13d | Describe any methods used to synthesize results and provide a rationale for the choice(s). If meta-analysis was performed, describe the model(s), method(s) to identify the presence and extent of statistical heterogeneity, and software package(s) used. | 7,8 |
|  | 13e | Describe any methods used to explore possible causes of heterogeneity among study results (e.g. subgroup analysis, meta-regression). | 7,8 |
|  | 13f | Describe any sensitivity analyses conducted to assess robustness of the synthesized results. | 7,8 |
| Reporting bias assessment | 14 | Describe any methods used to assess risk of bias due to missing results in a synthesis (arising from reporting biases). | 8 |
| Certainty assessment | 15 | Describe any methods used to assess certainty (or confidence) in the body of evidence for an outcome. | 8 |
| **RESULTS** | | |  |
| Study selection | 16a | Describe the results of the search and selection process, from the number of records identified in the search to the number of studies included in the review, ideally using a flow diagram. | 8,9 |
|  | 16b | Cite studies that might appear to meet the inclusion criteria, but which were excluded, and explain why they were excluded. | Supplementary File |
| Study characteristics | 17 | Cite each included study and present its characteristics. | 9,10 |
| Risk of bias in studies | 18 | Present assessments of risk of bias for each included study. | 10, 11, Supplementary File |
| Results of individual studies | 19 | For all outcomes, present, for each study: (a) summary statistics for each group (where appropriate) and (b) an effect estimate and its precision (e.g. confidence/credible interval), ideally using structured tables or plots. | Supplementary File |
| Results of syntheses | 20a | For each synthesis, briefly summarise the characteristics and risk of bias among contributing studies. | 11-13, Supplementary File |
|  | 20b | Present results of all statistical syntheses conducted. If meta-analysis was done, present for each the summary estimate and its precision (e.g. confidence/credible interval) and measures of statistical heterogeneity. If comparing groups, describe the direction of the effect. | 11-13 |
|  | 20c | Present results of all investigations of possible causes of heterogeneity among study results. | 11-13 |
|  | 20d | Present results of all sensitivity analyses conducted to assess the robustness of the synthesized results. | 11 |
| Reporting biases | 21 | Present assessments of risk of bias due to missing results (arising from reporting biases) for each synthesis assessed. | Table 1 |
| Certainty of evidence | 22 | Present assessments of certainty (or confidence) in the body of evidence for each outcome assessed. | 13,14, Table 2 |
| **DISCUSSION** | | |  |
| Discussion | 23a | Provide a general interpretation of the results in the context of other evidence. | 14-17 |
|  | 23b | Discuss any limitations of the evidence included in the review. | 16,17 |
|  | 23c | Discuss any limitations of the review processes used. | 17 |
|  | 23d | Discuss implications of the results for practice, policy, and future research. | 17,18 |
| **OTHER INFORMATION** | | |  |
| Registration and protocol | 24a | Provide registration information for the review, including register name and registration number, or state that the review was not registered. | 4 |
|  | 24b | Indicate where the review protocol can be accessed, or state that a protocol was not prepared. | 4 |
|  | 24c | Describe and explain any amendments to information provided at registration or in the protocol. | 4 |
| Support | 25 | Describe sources of financial or non-financial support for the review, and the role of the funders or sponsors in the review. | Submission system |
| Competing interests | 26 | Declare any competing interests of review authors. | Submission system |
| Availability of data, code and other materials | 27 | Report which of the following are publicly available and where they can be found: template data collection forms; data extracted from included studies; data used for all analyses; analytic code; any other materials used in the review. | - |

**Reference:**

Page MJ, McKenzie JE, Bossuyt PM, Boutron I, Hoffmann TC, Mulrow CD, et al. The PRISMA 2020 statement: an updated guideline for reporting systematic reviews. BMJ 2021;372:n71. doi: 10.1136/bmj.n71

# **Supplementary material 2.** Databases and respective search strategies employed, date of search and number of studies retrieved.

| **Databases** | **Date** | **Search strategy** | **Studies retrieved** |
| --- | --- | --- | --- |
| MEDLINE (PubMed) | 11/22/22 | (((((((Zolgensma[Supplementary Concept]) OR (Zolgensma[Text Word])) OR (onasemnogene abeparvovec-xioi[Text Word])) OR (AVXS 101[Text Word])) OR (onasemnogene abeparvovec xioi[Text Word])) OR (AVXS101[Text Word])) OR (AVXS-101[Text Word])) OR ("nusinersen"[Supplementary Concept] OR "nusinersen"[Text Word] OR "ASO-10-27"[Text Word] OR ("ISIS-SMN"[All Fields] AND "Rx"[All Fields]) OR "ISIS-SMNRx"[Text Word] OR "isis 396443"[Text Word] OR "SPINRAZA"[Text Word]) OR ((("Risdiplam" [Supplementary Concept]) OR (Risdiplam[Text Word])) OR (7-(4,7-Diazaspiro(2.5)oct-7-yl)-2-(2,8-dimethylimidazo(1,2-b)pyridazin-6-yl)-4H-pyrido(1,2-a)pyrimidin-4-one[Text Word])) AND ((Atrophy, Spinal Muscular[Text Word] OR Spinal Amyotrophy[Text Word] OR Amyotrophies, Spinal[Text Word] OR Amyotrophy, Spinal[Text Word] OR Spinal Amyotrophies[Text Word] OR Spinal Muscular Atrophy[Text Word] OR Distal Spinal Muscular Atrophy[Text Word] OR Spinal Muscular Atrophy, Distal[Text Word] OR Hereditary Motor Neuronopathy[Text Word] OR Hereditary Motor Neuronopathies[Text Word] OR Motor Neuronopathies, Hereditary[Text Word] OR Motor Neuronopathy, Hereditary[Text Word] OR Neuronopathies, Hereditary Motor[Text Word] OR Neuronopathy, Hereditary Motor[Text Word] OR Scapuloperoneal Form, Spinal Muscular Atrophy[Text Word] OR Spinal Muscular Atrophy, Scapuloperoneal Form[Text Word] OR Spinal Muscular Atrophy, Scapuloperoneal[Text Word] OR Amyotrophy, Neurogenic Scapuloperoneal, New England Type[Text Word] OR Scapuloperoneal Spinal Muscular Atrophy[Text Word] OR Oculopharyngeal Spinal Muscular Atrophy[Text Word] OR Spinal Muscular Atrophy, Oculopharyngeal[Text Word] OR Progressive Muscular Atrophy[Text Word] OR Atrophies, Progressive Muscular[Text Word] OR Atrophy, Progressive Muscular[Text Word] OR Muscular Atrophies, Progressive[Text Word] OR Muscular Atrophy, Progressive[Text Word] OR Progressive Muscular Atrophies[Text Word] OR Progressive Myelopathic Muscular Atrophy[Text Word] OR Myelopathic Muscular Atrophy, Progressive[Text Word] OR Progressive Proximal Myelopathic Muscular Atrophy[Text Word] OR Proximal Myelopathic Muscular Atrophy, Progressive[Text Word] OR Bulbospinal Neuronopathy[Text Word] OR Bulbospinal Neuronopathies[Text Word] OR Neuronopathies, Bulbospinal[Text Word] OR Neuronopathy, Bulbospinal[Text Word] OR Myelopathic Muscular Atrophy[Text Word] OR Atrophy, Myelopathic Muscular[Text Word] OR Muscular Atrophy, Myelopathic[Text Word] OR Adult-Onset Spinal Muscular Atrophy[Text Word] OR Adult Onset Spinal Muscular Atrophy[Text Word] OR Muscular Atrophy, Adult Spinal[Text Word] OR Adult Spinal Muscular Atrophy[Text Word]) OR (((Spinal Muscular Atrophies of Childhood[MeSH Terms]) OR (Spinal Muscular Atrophies of Childhood[Text Word])) OR (Muscular Atrophy, Spinal, Type II[Text Word] OR Spinal Muscular Atrophy Type 2[Text Word] OR Type II Spinal Muscular Atrophy[Text Word] OR Spinal Muscular Atrophy Type II[Text Word] OR Spinal Muscular Atrophy, Type II[Text Word] OR HMN (Hereditary Motor Neuropathy) Proximal Type I[Text Word] OR Spinal Muscular Atrophy, Infantile[Text Word] OR Muscular Atrophy, Spinal, Infantile[Text Word] OR Werdnig-Hoffmann Disease[Text Word] OR Werdnig Hoffmann Disease[Text Word] OR Muscular Atrophy, Spinal, Type I[Text Word] OR Spinal Muscular Atrophy Type I[Text Word] OR SMA, Infantile Acute Form[Text Word] OR Type I Spinal Muscular Atrophy[Text Word] OR Proximal Hereditary Motor Neuropathy Type I[Text Word] OR Werdnig Hoffman Disease[Text Word] OR Muscular Atrophy, Infantile[Text Word] OR Infantile Muscular Atrophy[Text Word] OR Spinal Muscular Atrophy 1[Text Word] OR Infantile Spinal Muscular Atrophy[Text Word] OR Spinal Muscular Atrophy, Type I[Text Word] OR Juvenile Spinal Muscular Atrophy[Text Word] OR Spinal Muscular Atrophy Type III[Text Word] OR Spinal Muscular Atrophy, Type III[Text Word] OR Type III Spinal Muscular Atrophy[Text Word] OR Muscular Atrophy, Juvenile[Text Word] OR Juvenile Muscular Atrophy[Text Word] OR Kugelberg-Welander Disease[Text Word] OR Kugelberg Welander Disease[Text Word] OR Spinal Muscular Atrophy, Type 3[Text Word] OR Kugelberg-Welander Syndrome[Text Word] OR Kugelberg Welander Syndrome[Text Word] OR Spinal Muscular Atrophy, Mild Childhood[Text Word] AND Adolescent Form[Text Word] OR Spinal Muscular Atrophy, Juvenile[Text Word] OR Muscular Atrophy, Spinal, Type III[Text Word] OR Muscular Atrophy, Spinal, Infantile Chronic Form[Text Word] OR Muscular Atrophy, Spinal, Intermediate Type[Text Word]))) | 663 |
| Embase | 11/22/22 | (('onasemnogene abeparvovec'/exp OR 'onasemnogene abeparvovec' OR 'avxs 101'/exp OR 'avxs 101' OR 'avxs101'/exp OR 'avxs101' OR 'charisma (drug)'/exp OR 'charisma (drug)' OR 'onasemnogene abeparvovec xioi'/exp OR 'onasemnogene abeparvovec xioi' OR 'onasemnogene abeparvovec-xioi'/exp OR 'onasemnogene abeparvovec-xioi' OR 'zolgensma'/exp OR zolgensma) AND [embase]/lim OR (('biib 058'/exp OR 'biib 058' OR 'biib058'/exp OR 'biib058' OR 'ionis smnrx'/exp OR 'ionis smnrx' OR 'ionis-smnrx'/exp OR 'ionis-smnrx' OR 'isis 396443'/exp OR 'isis 396443' OR 'isis smnrx'/exp OR 'isis smnrx' OR 'isis-smnrx'/exp OR 'isis-smnrx' OR 'isis396443'/exp OR 'isis396443' OR 'nusinersen'/exp OR 'nusinersen' OR 'nusinersen sodium'/exp OR 'nusinersen sodium' OR 'spinraza'/exp OR 'spinraza') AND [embase]/lim) OR (('2 (2, 8 dimethylimidazo [1, 2 b] pyridazin 6 yl) 7 (4, 7 diazaspiro [2.5] oct 7 yl) 4h pyrido [1, 2 a] pyrimidin 4 one'/exp OR '2 (2, 8 dimethylimidazo [1, 2 b] pyridazin 6 yl) 7 (4, 7 diazaspiro [2.5] oct 7 yl) 4h pyrido [1, 2 a] pyrimidin 4 one' OR '2 (2, 8 dimethylimidazo [1, 2 b] pyridazin 6 yl) 7 (4, 7 diazaspiro [2.5] octan 7 yl) 4h pyrido [1, 2 a] pyrimidin 4 one'/exp OR '2 (2, 8 dimethylimidazo [1, 2 b] pyridazin 6 yl) 7 (4, 7 diazaspiro [2.5] octan 7 yl) 4h pyrido [1, 2 a] pyrimidin 4 one' OR '7 (4, 7 diazaspiro [2.5] oct 7 yl) 2 (2, 8 dimethylimidazo [1, 2 b] pyridazin 6 yl) 4h pyrido [1, 2 a] pyrimidin 4 one'/exp OR '7 (4, 7 diazaspiro [2.5] oct 7 yl) 2 (2, 8 dimethylimidazo [1, 2 b] pyridazin 6 yl) 4h pyrido [1, 2 a] pyrimidin 4 one' OR '7 (4, 7 diazaspiro [2.5] octan 7 yl) 2 (2, 8 dimethylimidazo [1, 2 b] pyridazin 6 yl) 4h pyrido [1, 2 a] pyrimidin 4 one'/exp OR '7 (4, 7 diazaspiro [2.5] octan 7 yl) 2 (2, 8 dimethylimidazo [1, 2 b] pyridazin 6 yl) 4h pyrido [1, 2 a] pyrimidin 4 one' OR 'evrysdi'/exp OR 'evrysdi' OR 'rg 7916'/exp OR 'rg 7916' OR 'rg7916'/exp OR 'rg7916' OR 'risdiplam'/exp OR 'risdiplam' OR 'ro 7034067'/exp OR 'ro 7034067' OR 'ro7034067'/exp OR 'ro7034067') AND [embase]/lim)) AND (('chronic spinal muscular atrophy'/exp OR 'chronic spinal muscular atrophy' OR 'muscle atrophy, spinal'/exp OR 'muscle atrophy, spinal' OR 'muscular atrophy, spinal'/exp OR 'muscular atrophy, spinal' OR 'spinal muscle atrophy'/exp OR 'spinal muscle atrophy' OR 'spinal muscular atrophy'/exp OR 'spinal muscular atrophy' OR 'spine muscle atrophy'/exp OR 'spine muscle atrophy') AND [embase]/lim OR (('werdnig hoffmann disease'/exp OR 'werdnig hoffmann disease' OR 'hereditary progressive spinal muscular atrophy'/exp OR 'hereditary progressive spinal muscular atrophy' OR 'hereditary spinal progressive muscular atrophy'/exp OR 'hereditary spinal progressive muscular atrophy' OR 'hoffmann werdnig disease'/exp OR 'hoffmann werdnig disease' OR 'hoffmann werdnig syndrome'/exp OR 'hoffmann werdnig syndrome' OR 'infantile hereditary spinal muscular atrophy'/exp OR 'infantile hereditary spinal muscular atrophy' OR 'infantile muscular atrophy'/exp OR 'infantile muscular atrophy' OR 'infantile neurogenic muscle atrophy'/exp OR 'infantile neurogenic muscle atrophy' OR 'infantile progressive muscular atrophy'/exp OR 'infantile progressive muscular atrophy' OR 'infantile progressive spinal muscular atrophy'/exp OR 'infantile progressive spinal muscular atrophy' OR 'infantile spinal muscular atrophy'/exp OR 'infantile spinal muscular atrophy' OR 'progressive muscular atrophy, infantile'/exp OR 'progressive muscular atrophy, infantile' OR 'progressive spinal muscular atrophy, infantile'/exp OR 'progressive spinal muscular atrophy, infantile' OR 'spinal muscular atrophy type 1'/exp OR 'spinal muscular atrophy type 1' OR 'spinal muscular atrophy type i'/exp OR 'spinal muscular atrophy type i' OR 'type 1 spinal muscular atrophy'/exp OR 'type 1 spinal muscular atrophy' OR 'type i spinal muscular atrophy'/exp OR 'type i spinal muscular atrophy' OR 'werdnig hoffman disease'/exp OR 'werdnig hoffman disease' OR 'werdnig hoffmann paralysis'/exp OR 'werdnig hoffmann paralysis' OR 'werdnig hoffmann syndrome'/exp OR 'werdnig hoffmann syndrome' OR 'werdnig-hoffmann disease'/exp OR 'werdnig-hoffmann disease') AND [embase]/lim)) AND [embase]/lim NOT ([embase]/lim AND [medline]/lim) | 1024 |
| LILACS | 11/22/22 | ((mh:(Atrofia Muscular Espinal OR Muscular Atrophy, Spinal OR Atrofia Muscular Espinal)) OR (Atrofia Muscular Espinal Escapuloperoneal OR Atrofia Muscular Espinal Oculofaríngea OR Atrofia Muscular Espinal de Forma Escapulofibular OR Atrofia Muscular Espinal de Forma Escapuloperoneal OR Atrofia Muscular Espinhal OR Atrofia Muscular Espinhal Escapuloperoneal OR Atrofia Muscular Espinhal Oculofaríngea OR Atrofia Muscular Espinhal de Forma Escapulofibular OR Atrofia Muscular Espinhal de Forma Escapuloperoneal OR Atrofia Muscular Progressiva OR Neuronopatia Bulboespinhal OR exC10.228.854.468* OR exC10.574.562.500* OR exC10.668.467.500* OR Adult Onset Spinal Muscular Atrophy OR Adult Spinal Muscular Atrophy OR Adult-Onset Spinal Muscular Atrophy OR Amyotrophies, Spinal OR Amyotrophy, Neurogenic Scapuloperoneal, New England Type OR Amyotrophy, Spinal OR Atrophies, Progressive Muscular OR Atrophy, Myelopathic Muscular OR Atrophy, Progressive Muscular OR Atrophy, Spinal Muscular OR Bulbospinal Neuronopathies OR Bulbospinal Neuronopathy OR Distal Spinal Muscular Atrophy OR Hereditary Motor Neuronopathies OR Hereditary Motor Neuronopathy OR Motor Neuronopathies, Hereditary OR Motor Neuronopathy, Hereditary OR Muscular Atrophies, Progressive OR Muscular Atrophy, Adult Spinal OR Muscular Atrophy, Myelopathic OR Muscular Atrophy, Progressive OR Myelopathic Muscular Atrophy OR Myelopathic Muscular Atrophy, Progressive OR Neuronopathies, Bulbospinal OR Neuronopathies, Hereditary Motor OR Neuronopathy, Bulbospinal OR Neuronopathy, Hereditary Motor OR Oculopharyngeal Spinal Muscular Atrophy OR Progressive Muscular Atrophies OR Progressive Muscular Atrophy OR Progressive Myelopathic Muscular Atrophy OR Progressive Proximal Myelopathic Muscular Atrophy OR Proximal Myelopathic Muscular Atrophy, Progressive OR Scapuloperoneal Form of Spinal Muscular Atrophy OR Scapuloperoneal Spinal Muscular Atrophy OR Spinal Amyotrophies OR Spinal Amyotrophy OR Spinal Muscular Atrophy OR Spinal Muscular Atrophy, Distal OR Spinal Muscular Atrophy, Oculopharyngeal OR Spinal Muscular Atrophy, Scapuloperoneal OR Spinal Muscular Atrophy, Scapuloperoneal Form OR Atrofia Muscular Espinal Escapulofibular OR Atrofia Muscular Espinal Escapuloperoneal OR Atrofia Muscular Espinal Oculofaríngea OR Atrofia Muscular Progresiva OR Neuropatía Bulboespinal)) AND (zolgensma OR AVXS 101 OR AVXS‐101 OR onasemnogene abeparvovec-xioi OR onasemnogene abeparvovec OR OAV101 OR onasemnogeno abeparvoveque) | 0 |
| *Cochrane Library* | 11/22/22 | #1 (zolgensma):ti,ab,kw  #2 (AVXS 101):ti,ab,kw  #3 (AVXS‐101):ti,ab,kw  #4 (onasemnogene abeparvovec-xioi):ti,ab,kw  #5 (onasemnogene abeparvovec):ti,ab,kw  #6 MeSH descriptor: [Muscular Atrophy, Spinal] explode all trees  #7 #1 OR #2 OR #3 OR #4 OR #5  #8 #6 AND #7 | 02 |
| *Clinical trials* | 11/22/22 | Condition: Spinal Muscular Atrophy  Other terms: zolgensma OR AVXS 101 OR AVXS‐101 OR onasemnogene abeparvovec-xioi OR onasemnogene abeparvovec OR OAV101 | 19 |
| ICTRP | 11/22/22 | Condition: Spinal Muscular Atrophy  Intervention: zolgensma OR AVXS 101 OR AVXS‐101 OR onasemnogene abeparvovec-xioi OR onasemnogene abeparvovec OR OAV101 | 16 |

# **Supplementary material 3.** Characteristics of excluded studies.

| **Author, year** | **Title** | **Reason for exclusion** |
| --- | --- | --- |
| Al-Zaidy et al., 2018 | Health outcomes in spinal muscular atrophy type 1 following AVXS-101 gene replacement therapy | Reported outcomes of START study, better contemplated by the reports by Mendell et al., 2017 and Mendell et al., 2021 |
| Al-Zaidy et al., 2019 | AVXS-101 (Onasemnogene Abeparvovec) for SMA1: Comparative Study with a Prospective Natural History Cohort | Reported outcomes of START study, better contemplated by the reports by Mendell et al., 2017 and Mendell et al., 2021 |
| Brandsema et al., 2022 | Baseline Characteristics and Initial Safety Results in RESPOND: A Phase 4 Study of Nusinersen in Children with Spinal Muscular Atrophy (SMA) Who Received Onasemnogene Abeparvovec | Conference abstract |
| Chiriboga et al., 2022 | JEWELFISH: Safety, pharmacodynamic and exploratory efficacy data in non-naive patients with spinal muscular atrophy (SMA) receiving treatment with risdiplam | Conference abstract |
| Dabbous et al., 2019 | Event-free survival and motor milestone achievement following AVXS-101 and nusinersen interventions contrasted to natural history for type I spinal muscular atrophy patients | Conference abstract |
| Dabbous et al., 2022 | Real-world Outcomes of Nusinersen Alone and Onasemnogene Abeparvovec(OA) Alone or with Prior Nusinersen in Pediatric Patients with Spinal Muscular Atrophy (SMA): Interim Analysis of a US Retrospective Chart Review Study | Conference abstract |
| Dabbous, et al., 2019 | Survival, Motor Function, and Motor Milestones: Comparison of AVXS-101 Relative to Nusinersen for the Treatment of Infants with Spinal Muscular Atrophy Type 1 | Indirect comparison study of results of the START and ENDEAR studies |
| Darras et al., 2022 | Pooled Safety Data from the Risdiplam Clinical Trial Development Program | Conference abstract |
| Day et al., 2019 | Onasemnogene abeparvovec gene-replacement therapy (GRT) for spinal muscular atrophy type 1 (SMA1): Pivotal phase 3 study (STR1VE) update | Conference abstract |
| Day et al., 2021 | Long-term Follow-up (LTFU) of Onasemnogene Abeparvovec Gene Therapy in Spinal Muscular Atrophy (SMA) | Conference abstract |
| Finkel et al., 2019 | Intrathecal administration of AVXS-101 genereplacement therapy for sitting but non-ambulatory patients with spinal muscular atrophy (SMA): Phase 1/2a study (STRONG) | Conference abstract |
| Finkel et al., 2021 | Onasemnogene abeparvovec gene therapy for spinal muscular atrophy type 1: Phase 3 study (STR1VE-US) | Conference abstract |
| Lowes et al., 2017 | AmVXS-101 phase 1 gene therapy clinical trial in SMA type 1: Correlation between CHOP-INTEND and motor milestone achievements | Conference abstract |
| Lowes et al., 2019 | Impact of Age and Motor Function in a Phase 1/2A Study of Infants With SMA Type 1 Receiving Single-Dose Gene Replacement Therapy | Reported outcomes of START study, better contemplated by the reports by Mendell et al., 2017 and Mendell et al., 2021 |
| McMillan et al., 2021 | Onasemnogene Abeparvovec Gene Therapy in Presymptomatic Spinal Muscular Atrophy (SMA): SPR1NT Study Update in Children with 3 Copies of SMN2 | Conference abstract |
| Mendell et al., 2020 | Gene Therapy in Spinal Muscular Atrophy Type 1 (SMA1): Long-Term Follow-Up (LTFU) From the Onasemnogene Abeparvovec Phase 1 Clinical Trial | Conference abstract |
| Mendell et al., 2021 | Long-Term Follow-Up (LTFU) of onasemnogene abeparvovec gene therapy in spinal muscular atrophy (SMA) | Conference abstract |
| Mendell et al., 2021 | Long-term follow-up of the phase 1 start trial of onasemnogene abeparvovec gene therapy in spinal muscular atrophy type 1 | Conference abstract |
| Mercuri et al., 2019 | Onasemnogene abeparvovec gene-replacement therapy (GRT) for spinal muscular atrophy type 1 (SMA1): Global pivotal phase 3 study program (STR1VE-US, STR1VE-EU, STR1VE-AP) | Conference abstract |
| Mercuri et al., 2020 | Onasemnogene abeparvovec gene replacement therapy (GRT) for spinal muscular atrophy type 1 (SMA1): Pivotal phase 3 studies clinical update (STR1VE-EU and STR1VE-US) | Conference abstract |
| Mercuri et al., 2021 | Onasemnogene abeparvovec gene therapy for spinal muscular atrophy type 1 (SMA1): Phase III study update (STR1VE-EU) | Conference abstract |
| Mercuri et al., 2022 | Onasemnogene abeparvovec gene therapy for spinal muscular atrophy type 1: phase 3 study update (STR1VE-EU) | Conference abstract |
| Mirea et al., 2021 | Combination therapy with nusinersen and onasemnogene abeparvovec-xioi in spinal muscular atrophy type i | Population of interest treated with combined therapy only: ​​nusinersen and onasemnogen abeparvoveque |
| Muntoni et al., 2019 | Study design of STR1VE-EU, a phase 3 trial of AVXS-101 gene-replacement therapy (GRT) in patients with spinal muscular atrophy type 1 (SMA1) in Europe | Conference abstract |
| Muntoni et al., 2021 | Gene replacement therapy for symptomatic spinal muscular atrophy type 1: Final results of the Phase III STR1VE-EU study | Conference abstract |
| Rodriguez et al., 2022 | Efficacy and Safety of Onasemnogene abeparvovec in Spinal Muscular Atrophy: A Systematic Review and Meta-Analysis | Conference abstract |
| Servais et al., 2022 | Effectiveness and Safety of Onasemnogene Abeparvovecin Older Patients with Spinal Muscular Atrophy (SMA): Real-World Outcomes from the RESTORE Registry | Conference abstract |
| Servais et al., 2022 | Onasemnogene Abeparvovec Treatment Outcomes by Patient Weight at Infusion: Initial Findings from the RESTORE Registry | Conference abstract |
| Servais et al., 2022 | Safety and Effectiveness of Onasemnogene Abeparvovec Alone or with Other Disease-Modifying Therapies: Findings from RESTORE | Conference abstract |
| Shell et al., 2019 | Onasemnogene abeparvovec gene-replacement therapy (GRT) for spinal muscular atrophy type 1 (SMA1): Preliminary pulmonary and ventilatory findings from the phase 3 study (STR1VE) | Conference abstract |
| Shell et al., 2020 | Onasemnogene abeparvovec-xioi gene-replacement therapy for spinal muscular atrophy type 1: Pulmonary and ventilatory findings from the pivotal phase 3 us study (STR1VE) | Conference abstract |
| Sproule et al., 2017 | AmVXS-101 phase 1 gene therapy clinical trial in SMA type 1: Experience with pre-existing anti-AAV9 antibody in the SMA1 population | Conference abstract |
| Strauss et al., 2019 | Onasemnogene abeparvovec gene-replacement therapy (GRT) in presymptomatic spinal muscular atrophy (SMA): SPR1NT study update | Conference abstract |
| Strauss et al., 2019 | Onasemnogene abeparvovec-XIOI gene-replacement therapy in presymptomatic spinal muscular atrophy (SMA): SPR1NT study update | Conference abstract |
| Strauss et al., 2020 | Onasemnogene Abeparvovec-xioi Gene Therapy in Presymptomatic Spinal Muscular Atrophy (SMA): SPR1NT Study Update | Conference abstract |
| Strauss et al., 2020 | Onasemnogene abeparvovec gene-replacement therapy in presymptomatic spinal muscular atrophy: SPR1NT study update | Conference abstract |
| Strauss et al., 2021 | Onasemnogene abeparvovec gene therapy in presymptomatic spinal muscular atrophy (SMA): Spr1nt study update in children with 3 Copies of SMN2 | Conference abstract |
| Weiss et al., 2021 | Real-World Data for Onasemnogen Abeparvovec (Zolgensma) in Spinal Muscular Atrophy | Conference abstract |

# **Supplementary material 4.** Risk of bias assessment using the ROBINS-I tool for studies by Day et al. (2021); Mercuri et al. (2021) and Mendell et al. (2017).

| **Risk of bias domain** | **Assessments by outcome** | **Comment** | **Risk of bias judgement** |
| --- | --- | --- | --- |
| **Bias due to confounding** | Overall survival and event-free survival; motor function improvement; drug-related adverse events. | Studies do not have matching comparators, no masking, and possibly did not use appropriate analysis methods to control for confounders at baseline and during follow-up. | Critical |
| **Bias in selection of participants into the study** | Overall survival and event-free survival; motor function improvement; drug-related adverse events. | The selection of study participants did not appear to be related to intervention, outcome, or any prognostic factor. As the mean age of symptoms and diagnosis coincide, then, it is assumed that the intervention is applied in the same mean life span of the participants. | Low |
| **Bias in classification of interventions** | Overall survival and event-free survival; motor function improvement; drug-related adverse events. | Well-defined intervention. | Low |
| **Bias due to deviations from intended interventions** | Overall survival and event-free survival; motor function improvement; drug-related adverse events. | No information about deviation from the intended intervention, being implemented according to protocol. | Low |
| **Bias due to missing data** | Overall survival and event-free survival; motor function improvement; drug-related adverse events. | All results appear to have been reported and missing data were justified. | Low |
| **Bias in measurement of outcomes** | Overall survival and event-free survival. | Despite the evaluators' knowledge of the intervention received by the patients, it was considered that this factor would be inherent to a single-arm study and would hardly influence the effect of a hard outcome. | Moderate |
|  | Motor function improvement; drug-related adverse events. | The measurement was based on observation and subjective assessment of motor scores and on the reporting of adverse events, which can result in systematic errors in the measurement of outcomes. | Critical |
| **Bias in selection of the reported result** | Overall survival and event-free survival; motor function improvement; drug-related adverse events. | Reporting of studies in accordance with the protocols. | Low |

# **Supplementary material 5.** Clinical trial results for overall survival, event-free survival, motor and other milestones achievements.

| **Results** | **Clinical trials** | | | |
| --- | --- | --- | --- | --- |
|  | **START**  **(Mendell et al., 2017; 2021).** | | **STR1VE (Day et al., 2021)** | **STR1VE-EU (Mercuri et al., 2021)** |
| Follow-up time (mean) | 24 months | 120 months | 18 months^a^ | 18 months^a^ |
| Number of patients, n (%) | 12 (100) | 10 (100) | 22 (100) | 33 (100) ^b^ |
| Overall survival, n (%) | 12 (100) | 10 (100) | 21 (95) | 32 (97) |
| Event-free survival, n (%) | 12 (100) | 6 (60) | 20 (91) | 31/32 (97) |
| Motor and other milestones achievements, n (%) | | | | |
| speaks ‡ | 11 (92) | 11 (92)^c^ | NA | NA |
| swallows ‡ | 11 (92) | 11 (92)^c^ | 12 (55) | NA |
| brings hand to mouth‡ | 12 (100) | 12 (100)^c^ | NA | NA |
| controls head‡ | 11 (92) | 11 (92)^c^ | 17 (85) | 26 (78) |
| rolls over ‡ | 9 (75) | 9 (75)^c^ | 13 (59) | 19 (58) |
| crawls | 2 (17) | 2 (17)^c^ | 1 (5) | 1 (33) |
| sits with support † | 11 (92) | 11 (92)c | NA | NA |
| sits without support ≥ 5 seconds ‡ | 11 (92) | 11 (92)^c^ | NA | NA |
| sits without support ≥ 10 seconds § | 10 (83) | 10 (83)^c^ | 14 (64) | 15 (46) |
| sits without support ≥ 30 seconds ‡ | 9 (75) | 9 (75)^c^ | 13 (59) | NA |
| sits without support ≥ 30 seconds regardless of age ‡ | NA | NA | 14 (64) | 16 (49) |
| stands with assistance† | NA | 2 (20) | 1 (5) | 2 (6) |
| stands alone† | 2 (17) | 2 (17)^c^ | 1 (5) | 1 (3) |
| walks alone† | 2 (17) | 2 (17)^c^ | 1 (5) | 1 (3) |
| walks with assistance † | NA | NA | 1 (5) | 1 (3) |
| achieves a CHOP INTEND score ≥ 40 points | 11 (92) | 2 (17)* | 21 (95) | 24 (73) |
| achieves a CHOP INTEND score ≥ 50 points | 10 (83)* | 0 (0)* | 14 (64) | 14 (42) |
| achieves a CHOP INTEND score ≥ 60 points | 3 (25)* | 0 (0)* | 5 (23) | 1 (33)* |
| CHOP INTEND score increase from baseline, mean (SD) | | | | |
| at 1 month post-dosing | 9.8 | NA | 6.9  (SD 5.4) | 6.0  (SD 5.4) |
| at 3 months post-dosing | 15,4 | NA | 11.7  (SD 6.4) | 10.3  (SD 6.3) |
| at 6 months post-dosing | NA | NA | 14.6  (SD 7.0) | 13.6  (SD 6.6) |
| until the end of the study | 24.6 | NA | 26.1* | 21* |
| CHOP INTEND score |  |  |  |  |
| at end of the study | 52.8 | NA | 57.9* | 48.9* |

NA - outcome not assessed in the study.

SD - Standard Deviation.

^a^For the clinical outcomes of survival and event-free survival, follow-up refers to the age of 14 complete months, while for motor milestones it refers to the age of 18 complete months.

^b^ According to Mercuri et al. (2021), 32 of 33 patients completed the study and were included in the ITT population, but in this summary of results, we considered the total number of subjects included in the study.

^c^ According to Mendel et al. (2021), all motor milestones achieved in the START study were maintained, without regression or loss of function, over the 5-year extension.

‡ According to the Bayley Scales of Infant and Toddler Development.

† According to the Children’s Hospital of Philadelphia Infant Test of Neuromuscular Disorders (CHOP INTEND) scale.

§ According to the World Health Organization Multicentre Growth Reference Study scale.

* Data extracted from the study chart or corresponding appendix using the WebPlotDigitizer tool (<https://automeris.io/WebPlotDigitizer/>).

# **Supplementary material 6.** Clinical trial results for safety.

| **Results** | **Clinical trials** | | | |
| --- | --- | --- | --- | --- |
|  | **START**  **(Mendell et al., 2017; 2021).** | | **STR1VE**  **(Day et al., 2021)** | **STR1VE-EU**  **(Mercuri et al., 2021)** |
| Follow-up time (mean) | 24 months | 120 months | 18 months | 18 months |
| Number of patients, n (%) | 12 (100) | 10 (100) | 22 (100) | 33 (100)^a^ |
| Patients, n (%) | | | | |
| Any adverse event | 12 (100) | NR | 22 (100) | 32 (97) |
| Any serious adverse event | 10 (83) | 7 (70) | 10 (45) | 19 (58) |
| Drug-related adverse events | 3 (25) | - | 12 (55) | 24 (73) |
| Pyrexia | - | - | - | 4 (12) |
| Hypertransaminasaemia | - | - | - | 8 (24) |
| Constipation | - | - | - | 1 (3) |
| Gastroenteritis | - | - | - | 2 (6) |
| Alanine aminotransferase (ALT) increased | 3 (25) | - | 5 (23) | 7 (21) |
| Aspartate aminotransferase (AST) increased | 3 (25) | - | 6 (27) | 6 (18) |
| Hydrocephalus | - | - | 1 (5) |  |
| Vomiting | - | - | - | 3 (9) |
| Gastro-oesophageal reflux disease | - | - | - | 1 (3) |
| Hypertension | - | - | - | 1 (3) |
| Rhinovirus infection | - | - | - | 1 (3) |
| Viral infection | - | - | - | 1 (3) |
| Feeding disorder | - | - | - | 1 (3) |
| Hypernatraemia | - | - | - | 1 (3) |
| Thrombocytopenia | - | - | - | 1 (3) |
| Coagulation test abnormal | - | - | - | 1 (3) |
| Serious-drug related adverse events | NA | 0 (0) | 3 (14) | 6 (18) |
| Alanine aminotransferase (ALT) increased | NA | - | 1 (5) | 1 (3) |
| Aspartate aminotransferase (AST) increased | NA | - | 1 (5) | 1 (3) |
| Hydrocephalus | NA | - | 1 (5) | NR |
| Pyrexia | NA | - | - | 2 (6) |
| Hypertransaminasaemia | NA | - | - | 1 (3) |
| Gastroenteritis | NA | - | - | 1 (3) |
| Rhinovirus infection | NA | - | - | 1 (3) |
| Viral infection | NA | - | - | 1 (3) |
| Feeding disorder | NA | - | - | 1 (3) |
| Hypernatraemia | NA | - | - | 1 (3) |
| Thrombocytopenia | NA | - | - | 1 (3) |
| Coagulation test abnormal | NA | - | - | 1 (3) |

NA - outcome not assessed in the study.

^a^ According to Mercuri et al. (2021), 32 of 33 patients completed the study and were included in the ITT population, but in this summary of results, we considered the total number of subjects included in the study.

# **References**

Mendell J. R., et al. (2017). Single-dose gene-replacement therapy for spinal muscular atrophy. New England Journal of Medicine, 377(18), 1713-1722.

Mendell, J. R., et al. (2021). Five-year extension results of the phase 1 START trial of onasemnogene approves in spinal muscular atrophy. JAMA neurology, 78(7), 834-841.

Day, J. W., et al. (2021). Onasemnogene abeparvovec gene therapy for symptomatic infantile-onset spinal muscular atrophy in patients with two copies of SMN2 (STR1VE): an open-label, single-arm, multicentre, phase 3 trial. The Lancet Neurology, 20(4), 284-293.

Mercuri E, et al. Onasemnogene abeparvovec gene therapy for symptomatic infantile-onset spinal muscular atrophy type 1 (STR1VE-EU): an open-label, single-arm, multicentre, phase 3 trial. Lancet Neurol 2021; 20: 832–41.
